# Synthesis and Characterization of β-Cyclodextrin-Hybridized Exfoliated Kaolinite Single Nanosheets as Potential Carriers of Oxaliplatin with Enhanced Loading, Release, and Cytotoxic Properties

**DOI:** 10.3390/ma16144958

**Published:** 2023-07-12

**Authors:** Mashael D. Alqahtani, Nourhan Nasser, May N. Bin Jumah, Saleha A. AlZahrani, Ahmed A. Allam, Mostafa R. Abukhadra, Stefano Bellucci

**Affiliations:** 1 Department of Biology, College of Science, Princess Nourah bint Abdulrahman University, P.O. Box 84428, Riyadh 11671, Saudi Arabia; mdalqahtani@pnu.edu.sa (M.D.A.);; 2 Geology Department, Faculty of Science, Beni-Suef University, Beni-Suef 65211, Egypt; 3 Materials Technologies and Their Applications Lab, Geology Department, Faculty of Science, Beni-Suef University, Beni-Suef 65211, Egypt; 4 Zoology Department, Faculty of Science, Beni-Suef University, Beni-Suef 65211, Egypt; 5 INFN-Laboratori Nazionali di Frascati, Via E. Fermi 54, 00044 Frascati, Italy

**Keywords:** kaolinite, exfoliation, β-cyclodextrin, oxaliplatin, loading, cytotoxicity

## Abstract

Natural kaolinite was subjected to a successful exfoliation process into separated kaolinite nanosheets (KNs), followed by hybridization with β-cyclodextrin biopolymer (β-CD), forming an advanced bio-nanocomposite (β-CD/KNs). The synthetic products were evaluated as enhanced delivery structures for oxaliplatin chemotherapy (OXAPN). The hybridization of KNs with β-CD polymer notably enhanced the loading capacity to 355.3 mg/g (β-CD/KNs) as compared to 304.9 mg/g for KNs. The loading of OXAPN into both KNs and β-CD/KNs displayed traditional pseudo-first-order kinetics (R^2^ > 0.85) and a conventional Langmuir isotherm (R^2^ = 0.99). The synthetic β-CD/KNs validates a greater occupied effective site density (98.7 mg/g) than KNs (66.3 mg/g). Furthermore, the values of the n steric parameter (4.7 (KNs) and 3.6 (β-CD/KNs)) reveal the vertical orientation of the loaded molecules and the loading of them by multi-molecular mechanisms. These mechanisms are mainly physical processes based on the obtained Gaussian energy (<8 KJ/mol) and loading energy (<40 KJ/mol). The release profiles of both KNs and β-CD/KNs extend for about 120 h, with remarkably faster rates for β-CD/KNs. According to the release kinetic findings, the release of OXAPN displays non-Fickian transport behavior involving the cooperation of diffusion and erosion mechanisms. The KNs and β-CD/KNs as free particles showed considerable cytotoxicity and anticancer properties against HCT-116 cancer cell lines (71.4% cell viability (KNs) and 58.83% cell viability (β-CD/KNs)). Additionally, both KNs and β-CD/KNs significantly enhanced the OXAPN’s cytotoxicity (2.04% cell viability (OXAPN/KNs) and 0.86% cell viability (OXAPN/β-CD/KNs).

## 1. Introduction

The majority of mortality globally has been attributed to noncontagious illnesses, notably the most prevalent types of cancer, and this effect is predicted to expand by 75% in the coming years [1,2]. Approximately 13% of cancer patients around the world suffer from colorectal cancer, which is among the most prevalent tumors of the gastrointestinal system [3,4]. It is one of the two major leading factors that cause death and raise mortality rates throughout the world and has a remarkable negative impact on human life. Colorectal cancer originates as polyps in the mucosal tissues and then expands to the submucosa and its surrounding tissues. Then, during the cancer’s most advanced stages, the oncologic cells that were generated deeply invade the lymph nodes and surrounding organs [5,6,7]. A variety of chemotherapies have been established to mitigate the proliferation of cancerous cells [8,9]. However, the majority of commonly used chemotherapies are cytotoxic to healthy, fresh cells and have quite a number of negative effects on a wide range of organs, including kidney failure and the suppression of bone marrow [4,5]. Several techniques have been studied to improve the selectivity and biological safety of the majority of frequently used chemotherapies [5]. This includes developing novel forms of chemotherapeutics or increasing the curative and safety properties of widely prescribed drugs to fulfill worldwide demand and the high cost of living in developing and poor countries [3].

Several studies show that oxaliplatin (OXAPN) is one of the most powerful cancer chemotherapy drugs used to treat malignant tumors. This is because it makes platinum-based chemical complexes that strongly stop DNA from dividing in tumor cells [3,4,10]. However, although the FDA organization permitted the OXAPN drug to be used as a chemotherapeutic during the course of therapy for tumors that have metastasized to other tissues, its metabolites and related derivatives have a significant toxic effect on both normal and tumor cells [7,11]. Myelotoxicity, cardiotoxicity, and gastrointestinal issues are only a few of the major side effects that OXAPN displays throughout the treatment period [7,12]. In addition, other issues regarding OXAPN’s low bloodstream solubility have been observed [6,13]. As a result, numerous novel delivery methods have been established as promising techniques to enhance the specificity, solubility, therapeutic significance, and release rate of OXAPN and can additionally preserve the given dosage of the drug at appropriate levels [11,12]. This may reduce the medicine’s negative effects on normal cells while also regulating how long malignant cells stay exposed to the drug ions [9,11].

A variety of inorganic, organic, and organic/inorganic blended structures have successfully delivered the OXAPN medication [9,14,15]. These structures significantly enhance the retention and penetration properties of anticancer medications. Alginate, cellulose/zeolite, polymers, cyclodextrin/phillipsite, mesoporous silica, lipid nanoparticles, bentonite/cellulose, and liposomes are all used in these formulas [4,9,11,12]. It has been generally documented that the most efficient carriers of the majority of chemotherapies are clay minerals such as kaolinite, sepiolite, vermiculite, halloysite, and montmorillonite. A wide variety of clay minerals possess distinctive layered aluminosilicate structures that have notable ion exchange potential, biocompatibility, non-toxicity, cost-effectiveness, adsorption capacity, heat resistance, and versatile chemical characteristics [16,17,18,19].

Kaolinite is a clay mineral that naturally forms with a chemical structure of hydrous aluminum silicate with 1:1 layered tetrahedron/octahedron units [12,20]. Although kaolinite is common in nature and is relatively affordable in comparison with other industrial clay minerals like montmorillonite, there has not been enough investigation into the mineral’s potential as a pharmaceutical delivery structure [12,21]. This is because it was thought to have a small surface area, release drugs quickly, have a weaker ability to exchange ions, and not be able to absorb drugs as well as bentonite and halloysite, the two most common clay-based drug carriers [22]. Therefore, a variety of modification techniques, involving organic functionalization, exfoliation, polymeric intercalation, scrolling, and inorganic surface functionalization, have been successfully used to further enhance both the chemical and physical characteristics of kaolinite.

The morphological features of the synthesized materials have an extensive impact on their biological, chemical, and physical characteristics; the morphology may substantially affect their surface area, adsorption capacities, and quantity of exposed active sites [23]. The fabrication of nanomaterials with two-dimensional and one-dimensional geometric forms has been recommended for a wide range of industrial applications due to their brilliant surface area, high dispersion characteristics, and notable surface reactive properties [24,25,26]. The exfoliation of the multilayered silicate units of clay minerals into separate forms of individual silicate layers with 2D forms has been established as a sophisticated modification technology in the last few years. This technique has been used successfully to develop novel clay nanostructures that have excellent biological compatibility, oxidization characteristics, adsorption capacity, surface reactivity, anticancer properties, surface area, and dispersion qualities [12,27]. However, this strategy was extensively investigated for montmorillonite, whereas exfoliating kaolinite and its functionalized product has only been covered by a few studies [12,27].

Furthermore, the polymer hybridization of the surfaces of silicates with prevalent biopolymers results in novel blended structures with extensively improved organophilic characteristics, biocompatibility, loading, biodegradability, and release properties [4,28,29,30]. β-cyclodextrin (β-CD) is a widely used and significant biopolymer that has been extensively investigated as an essential component of numerous synthetic composites with a variety of inorganic materials for various pharmaceutical and environmental uses [31,32]. This was attributed mostly to its chemical stability, availability, adsorption properties, biocompatibility, and biosafety [33,34]. Chemically, β-CD has a cyclic glucopyranose framework consisting of six or seven glucose units, which are bonded together via a number of α (1 → 4) glycosidic bonds [32,35]. While the outside surfaces of these units involving their essential chemical groups possess substantial polarity, the interior structures of β-CD have a hydrophobic nature [36]. This greatly promotes their incorporation into nanocomposites that comprise inorganic materials and maximizes their synthetic carriers’ capacity to load drugs [33,37]. Moreover, the integrated β-CD substantially improves the drug’s physicochemical characteristics, including solubility in water, therapeutic effectiveness, chemical stability, and physical stability, when employed as a carrier for drugs or delivery systems [38].

Therefore, the presented study involved, for the first time, the morphological alteration of kaolinite into separated single nanosheets and the functionalization of the product with β-CD polymeric chains (β-CD/KNs) to produce a hybrid structure that possesses significant organophilic characteristics as a potential carrier of oxaliplatin with better loading, release, and anticancer activity. The OXAPN-loading characteristics were assessed depending on the basic experimental variables, and the influencing mechanisms were demonstrated via traditional and advanced equilibrium models. Furthermore, the release profiles are described in depth, and the key mechanisms are illustrated based on the mathematical findings of the release kinetic investigations. Moreover, the cytotoxic characteristics of free KNs and β-CD/KNs as well as their OXAPN-loading products were assessed to detect their inhibition effects on colorectal cancer cells (HCT-116).

## 2. Experimental Work

### 2.1. Materials

The kaolinite powder incorporated during the synthesis steps of separated kaolinite single nanosheets was used as a refined sample and delivered directly from the Central Metallurgical and Development Institute in Egypt. Dimethyl sulfoxide (DMSO) (>99.5%; Sigma-Aldrich; Cairo, Egypt), cetyltrimethylammonium bromide (CTAB) (>98%; Sigma-Aldrich; Egypt), and methanol (>99.9%; Sigma-Aldrich; Egypt) were incorporated during the exfoliation processes. High-purity β-cyclodextrin polymer (>85%; MW 1153 g/mol; Sigma-Aldrich; Egypt), in addition to high-purity ethanol (95%; Sigma-Aldrich; Egypt), was applied during the polymeric hybridization reactions. Oxaliplatin (Sigma-Aldrich; MW 397.29) was used during the loading and release tests.

### 2.2. Synthesis of Kaolinite Nanosheets (KNs)

**Figure 1:**
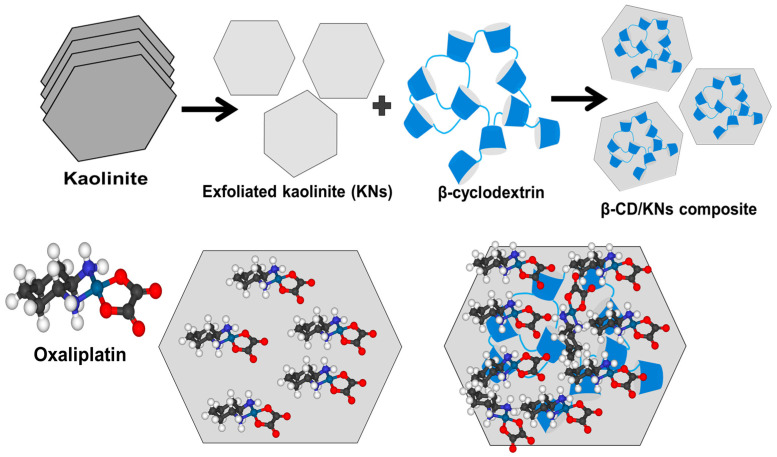
Schematic diagram for the synthesis of KNs and β-CD/KNs and their loading with OXAPN molecules.

A straightforward chemical expansion technique exfoliated the kaolinite-layered units. Kaolinite was ground into a powder with a size range of 20 to 100 nm by being ground in a ball mill for about 6 h. After that, 15 g of powdered kaolinite was mixed for 5 h with 50 mL of a diluted DMSO solution (8 (DMSO):1 (distilled water)) using a standard magnetic stirring device. This procedure is necessary to break down the hydrogen bonds that connect the multilayered silicate units of the mineral kaolinite together. Then, the kaolinite that had been treated with DMSO was rinsed with methanol for 20 min. This process was repeated five times to wash away all of the DMSO molecules and replace them with alcohol molecules, making a material called methoxy kaolinite, which is attracted to water. The methoxy kaolinite particles were then mixed together in a CTAB solution (20 g CTAB + 50 mL distilled water) that had already been made. This took 48 h and used a magnetic stirrer and a high-frequency ultrasound source (240 W) as part of an integrated mixing system. This caused the kaolinite to separate into single nanosheets (KNs). The produced KN particles were subsequently thoroughly washed with distilled water, gently dried for 12 h at 65 °C, and ascribed to KNs (Figure 1).

### 2.3. Synthesis of β-Cyclodextrin/KNs (β-CD/KNs)

According to the described procedure by Altoom et al. [4], the hybridization process of KN nanoparticles with β-CD was completed. A slurry-like solution was produced by dispersing and homogenizing the β-CD powders (1 g) with approximately 80 mL of ethanol over 3 h using a magnetic stirrer (1000 rpm). As part of a cooperative experiment, 2 g of previously synthesized KN particles was further transferred and homogenized for 60 min in 100 mL of distilled water while being stirred at 1000 rpm and sonicated at 240 W. Following that, the KN slurry that was produced and the water-based solution of β-CD were blended together, and the mixing was accomplished by stirring (at 1000 rpm) for 24 h. Then, the leftover solution was homogenized again for 24 h using an ultrasound source (240 W). After that, the hybrid particles were carefully separated from the solution using Whatman filter paper (40 µm). The obtained product was rinsed with distilled water to neutralize its surface and dried at 60 °C for 12 h before being used in the subsequent experiments (Figure 1).

### 2.4. Analytical Techniques

Depending on the observed XRD patterns, the crystallinity degree and crystal phases were determined using a PANalytical-Empyrean X-ray diffractometer within the measuring range of 0 to 70°. A Fourier transform infrared spectrometer (FTIR8400S; Shimadzu, Kyoto, Japan) was used to distinguish between the chemical structures of KNs and β-CD/KNs in addition to the prepared intermediate materials. The detection frequency range for this measurement was 400 cm^−1^ to 4000 cm^−1^. The SEM images were acquired via a scanning electron microscope (Gemini, Zeiss Ultra 55, Oberkochen, Germany), and shortly afterward the synthetic clay structures were coated with thin films of gold. SEM imaging was used to verify the expected modifications in the morphological characteristics of kaolinite throughout the various transformation processes. Additionally, the HRTEM images that were acquired using a transmission electron microscope (JEOL-JEM2100, Tokyo, Japan) at a 200 kV accelerating voltage were used to investigate the interior properties of KNs as well as β-CD/KNs. The surface area and porosity of KNs and β-CD/KNs were assessed using a surface area analyzer (Beckman Coulter SA3100, Indianapolis, IN, USA) based on the corresponding N_2_ adsorption/desorption isotherms.

### 2.5. OXAPN-Loading Studies

The OXAPN encapsulation potential of KNs and β-CD/KNs as delivery structures was investigated and studied according to a variety of variables that control the loading capacity. As affecting loading factors, the loading pH (2–9), duration of loading (1–24 h), tested OXAPN concentration (100–800 mg/L), and temperature (20–60 °C) were all investigated. After considering all the different variables influencing the reactions, a vortex rotator apparatus was applied during the loading procedures to homogeneously mix the KN and β-CD/KNs particles within 50 mL of the OXAPN solutions. Following the end of each loading experiment, the supplemented particulates of KNs and β-CD/KNs were separated from the OXAPN solutions using Whatman filter papers. The remaining OXAPN ions within the filtrates were monitored utilizing a UV-Vis spectrophotometer (λ_(max)_ = 209 nm), and their measured concentrations were subsequently utilized to compute the loading capacity or loaded quantities in mg/g according to Equation (1). All loading tests were performed in three distinct tests, and the declared OXAPN concentrations are provided as averages (Figure 1).

(1) $$Loaded\;drug\left(mg/g\right)=\frac{\left(Initial\;concentration-Residual\;concentration\right)\times solvent\;volume}{Carrier\;weight}$$

### 2.6. The Release Studies

The release characteristics and diffusion profiles of OXAPN from both KNs and β-CD/KNs were tracked using two releasing media that had various pH values (phosphate-buffered saline solutions (pH 7.4) and acetate-buffered saline solutions (pH 5.5)) at a constant temperature of 37.5 °C. The loaded KN and β-CD/KNs carriers (100 mg of OXAPN) were homogenized in 500 mL of each of the abovementioned buffers in two separate experiments. Using DISTEK dissolving equipment with a preset rotation speed of 200 rpm, the mixture homogenization process was completed in 180 h. Based on periodically obtained samples (5 mL) from both of the utilized buffers, the exact levels of the liberated OXAPN were determined by a UV-Vis spectrophotometer (λ_(max)_ = 209 nm). The samples that were periodically extracted were then reinserted into the whole releasing buffers to ensure that the process occurred under the same circumstances. This was performed three times, and the mean values were used to compute the released percentages using Equation (2).

(2) $$Drug\;release(\%)=\frac{The\;amount\;of\;Released\;OXAPN}{Amount\;of\;loaded\;OXAPN}\times 100$$

### 2.7. In Vitro Cytotoxicity

#### 2.7.1. Cell Lines

Colorectal cancer cell lines (HCT-116) were delivered from the American Type Culture Collection (ATCC, Rockville, MD, USA) and incorporated as cancerous cells during the cytotoxic assays. Trypsin-EDTA (0.25%), gentamycin, dimethyl sulfoxide (DMSO), fetal bovine serum, 3(4, 5-dimethylthiazol-2-yl)-2.5 diphenyltetrazolium bromide (MTT 99%), HEPES buffer, DMEM, and RPMI-1640 were the main reagents that were used either during the incubation process or the cytotoxic assay. All the incubation processes and the cytotoxicity assays were carried out at the Regional Center for Mycology and Biotechnology, Al-Azhar University, Cairo, Egypt.

#### 2.7.2. In Vitro Cytotoxicity

First, the malignant HCT-116 cell lines were cultured in RPMI-1640 media containing 50 µg/mL gentamycin and 10% fetal calf serum at 37 °C and 5% CO_2_. After a three-week culture period, the malignant cell lines (5 × 10^4^ cells/well) were submerged in Corning^®^ 96-well plates for 24 h. Following this, specific amounts of the OXAPN-loaded K, KN, and KNT carriers (100 mg of OXAPN) were delivered to each cell strain, and afterward, they were cultivated again for an additional 5 days. The loaded samples were prepared according to the estimated best loading conditions (pH 8, 20 °C (temperature), 50 mL (solution volume), 100 mg/L drug concentration, and 24 h (duration)). The quantity of viable cells produced throughout the course of incubation was measured using the commonly used MTT cell proliferation assay. By completing the incubation cycle, the integrated culture medium was effectively removed and replaced with freshly developed media (100 µL of RPMI). The newly added medium was well-mixed with the MTT (10 µL; 12 mM), and the mixture was then cultured again for 5 h until the notable growth of formazan with a recognizable purple color. After that, 50 µL of DMSO solution was used to effectively dissolve the formed formazan. The last step involves determining the optical density of the cell lines cultured during the studies using a microplate at a specific wavelength of 590 nm. The calculated values were used to compute cell viability% according to Equation (3).

(3) $$Cell\;viability\left(\%\right)=\frac{Mean\;OD}{Control\;OD}\times 100$$

## 3. Results and Discussion

### 3.1. Characterization of the Carrier

#### 3.1.1. XRD Analysis

**Figure 2:**
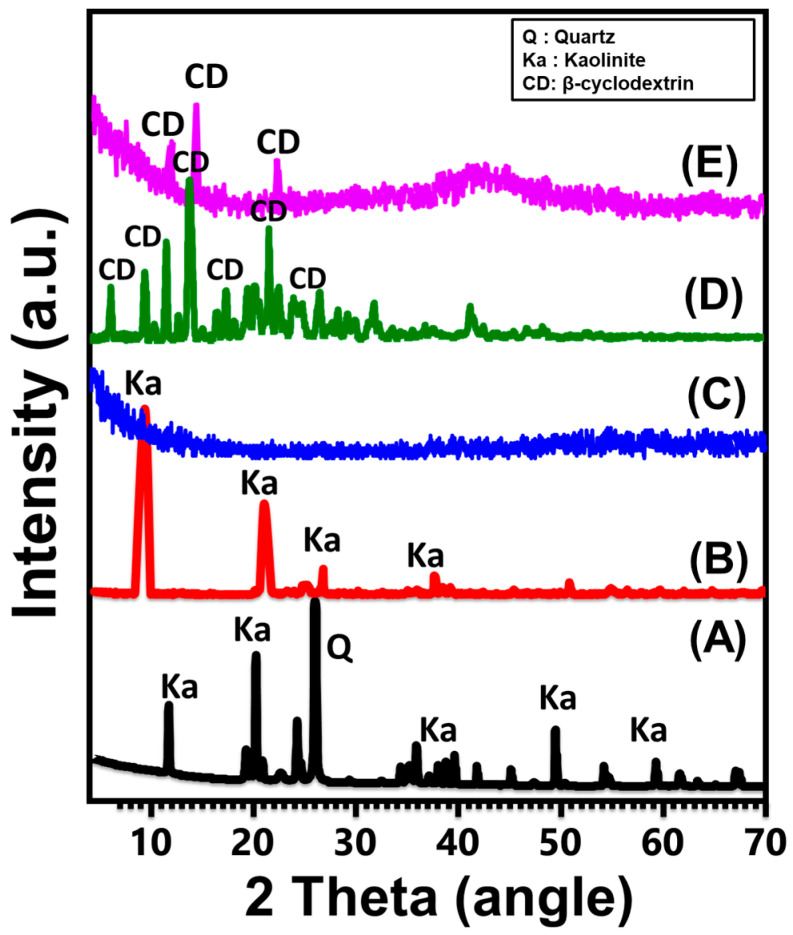
XRD patterns of raw kaolinite (A), DMSO-modified kaolinite (B), exfoliated kaolinite (C), β-cyclodextrin polymer (D), and synthetic β-CD/KNs composite (E).

The structural changes from the crystalline characteristics of kaolinite raw materials to single kaolinite nanosheets (KNs) and β-CD/KNs composites were monitored according to XRD patterns. The characteristic peaks of triclinic and well-crystallized kaolinite with its d-spacing value (0.72 nm) can be observed clearly in the pattern of the starting mineral at 12.33° (001), 20.85° (−110), 24.87° (002), and 26.64° (111) (Figure 2A). The total number of distinctive kaolinite diffraction peaks was greatly reduced after the DMSO incorporation stage, with a few exceptions of the corresponding peaks (001) and (002), which were greatly shifted (Figure 2B). The diffraction pattern that was detected after the sonication-induced exfoliation reactions (KNs) revealed a complete reduction for all leftover peaks, and the transformed material seemed to exhibit an amorphous property (Figure 2C). This demonstrates that the multi-layered kaolinite units were effectively separated into individual non- or semi-crystalline silicate sheets.

The strong crystalline characteristics of the integrated polymer precursor are reflected in its diffraction pattern, which exhibits numerous intensive diffraction peaks at 2 Theta angles of 6.80°, 9.20°, 10.83°, 12.57°, 12.64°, 12.80°, 13.0°, 15.55°, 18.90°, 19.80°, 21.90°, 23.00°, 25.81°, 27.34°, and 30° (Figure 2D). The observed pattern of β-CD/KNs shows the presence of the XRD peaks of β-cyclodextrin, but at noticeably deviating sites, there is a considerable decrease in intensity and a considerably increased broadness of those peaks (Figure 2E). This pattern reveals the integration and modification of KNs with β-cyclodextrin chains, which may occur by grafting the β-CD chains, generating chemical complexes, or forming hydrogen bonds. Moreover, the hybridization process is associated with the amorphization impact of the β-CD chains, which might enhance their reactivity (Figure 2E).

#### 3.1.2. SEM and HRTEM Analyses

**Figure 3:**
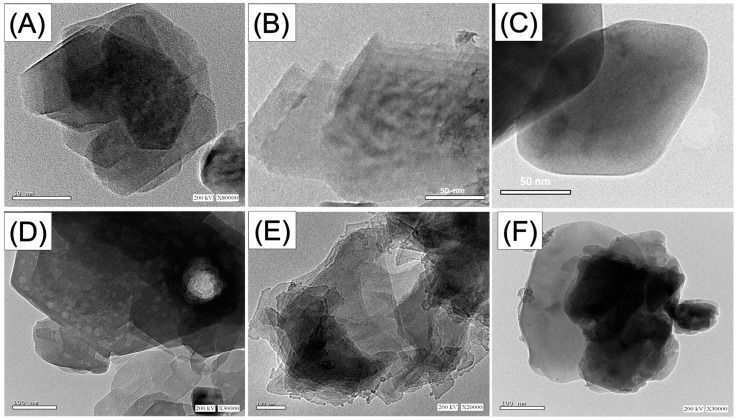
HRTEM image of raw kaolinite (**A**), synthetic exfoliated kaolinite sheets (**B**–**D**), and synthetic β-CD/KNs composite (**E**,**F**).

In terms of the morphological changes that occurred throughout the various synthesis processes, the incorporated raw kaolinite particles displayed the characteristic packed pseudo-hexagonal flaky or platey-like granules (Figure 3A). The HRTEM photos of the exfoliated products demonstrate that the kaolinite mineral was extensively peeled away and split into individual layers (Figure 3B). Other investigated images demonstrated the existence of exfoliated single sheets with smoother borders than the fresh kaolinite flakes; however, they still maintained a general pseudo-hexagonal form (Figure 3C). Some investigated images show lighter gray tones compared to the general gray tone of the peeled kaolinite sheets, indicating disorder in the silicate units of the kaolinite structure (Figure 3D). After the integration of the obtained kaolinite single sheets with β-CD polymer, there were also noticeable changes in the morphological features (Figure 3E,F). The HRTEM images of the obtained particles of the β-CD/KNs composite appeared in agglomerated forms, demonstrating the interaction effect of the blocky matrix of β-CD as cement material for the kaolinite sheets (Figure 3E). The high-magnification HRTEM images significantly reflect the enclaves of the kaolinite sheets within the matrix of β-CD polymer (Figure 3F). The morphological transformation of kaolinite is associated with a remarkable enhancement in the determined surface area as the value of raw kaolinite (10 m^2^/g) increased to 80.2 m^2^/g and 85.7 m^2^/g after the exfoliation (KNs) and the integration with β-CD polymer (β-CD/KNs), respectively.

#### 3.1.3. FT-IR Analysis

**Figure 4:**
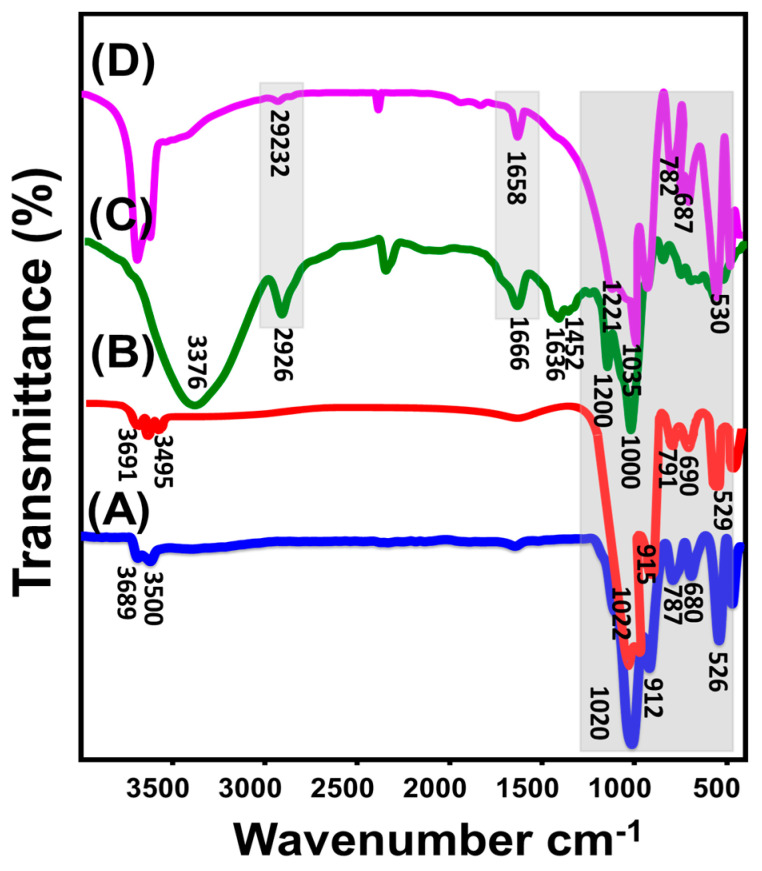
FT–IR spectra of raw kaolinite (A), exfoliated kaolinite (B), β-cyclodextrin polymer (C), and synthetic β-CD/KNs composite (D).

According to their FT-IR spectra, the influence of the various modification processes used to convert kaolinite into KNs and β-CD/KNs on the functional chemical groups was evaluated. The distinctive groups of kaolinite’s aluminosilicate framework, such as Si-O (787 and 456 cm^−1^), Si-O-Al (526 and 680 cm^−1^), Si-O-Si (1020 cm^−1^), Al-OH (912 and 3500 cm^−1^), O-H (1641 cm^−1^), and Si-OH (3689 cm^−1^), are clearly discernible in the mineral’s spectrum [20,39] (Figure 4A). The detected spectrum of KNs displays precisely similar absorption bands as those identified in raw kaolinite, but with significant shifts in their positions, a decrease in their intensities, and the observable splitting of recognizable bands around 900 cm^−1^ and 1000 cm^−1^ (Figure 4B). This indicates that the aluminosilicate layers of kaolinite were effectively exfoliated into monolayer sheets or separated layers and suggests potential distortions of the kaolinite’s octahedron and tetrahedron units [22,40] (Figure 4B).

The observed spectrum of β-cyclodextrin precisely demonstrates its basic chemical groups that involve the polysaccharides and glycosidic binding, such as O-H stretching vibration (3376 cm^−1^), –CH/CH_2_ asymmetrical stretching (2926 cm^−1^), the stretching mode of C-C and/or H-O-H deformation within the β-CD cavity (1666.2 cm^−1^), C=O stretching and/or OH bending (1636 cm^−1^), C-OH bending vibration (1482 cm^−1^), symmetrical C-O-C (1200 cm^−1^), asymmetrical C–O–C stretching (1158 cm^−1^), and symmetrical C-O stretching (1000 cm^−1^) (Figure 4C) [1,36]. The effective functionalization of KNs with the polymer chains of β-CD manifested itself in the assessed FT-IR spectrum of the composite (Figure 4D). Some of the identifying absorption bands of β-CD (asymmetrical –CH/CH_2_ (2932 cm^−1^), C-C (1658.6 cm^−1^), and symmetrical C-O-C (1221 cm^−1^)) can be noticed in the perceived spectrum, in addition to the distinctive absorption bands of KNs (Si-O (782 and 461 cm^−1^), Si-O-Al (530 and 687 cm^−1^), Si-O-Si (1035 cm^−1^), Al-OH (918 and 3500 cm^−1^), and Si-OH (3690 cm^−1^)) (Figure 4D). Therefore, the formation of β-CD/KNs can be verified by the recognition of complex organic/inorganic chemical groups as well as by the noticeable fluctuating positions of the characterization absorption bands.

### 3.2. Encapsulation of OXAPN Drug

#### 3.2.1. Influence of the Encapsulation Parameters

##### Effect of pH

**Figure 5:**
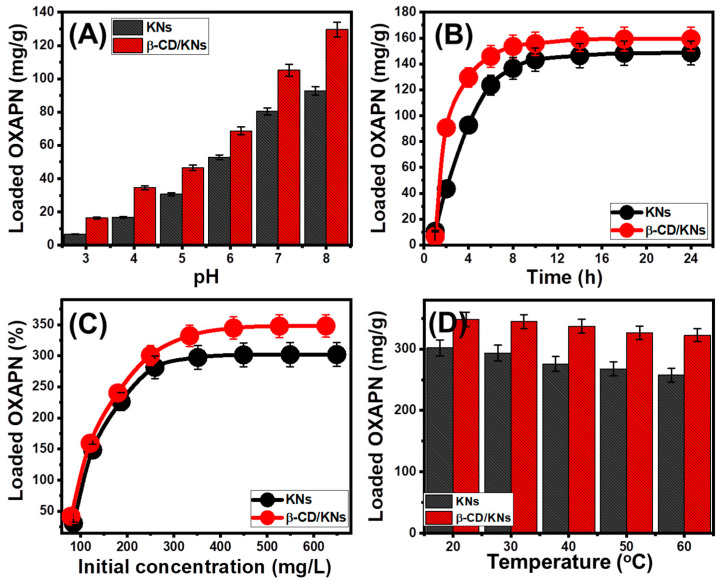
Effect of the experimental variables on the loading of OXAPN into KNs and β-CD/KNs including the pH (**A**), loading duration (**B**), OXAP concentration (**C**), and loading temperature (**D**).

The ionization state of the medication and the predominant charges on the carriers’ surfaces are both influenced by the pH value of the solution being used. This has a substantial effect on the extent to which the OXAPN can be encapsulated into KNs and β-CD/KNs. The impact of pH was assessed from pH 3 to pH 8 under the specific values of the main experimental variables (25 mg (carrier dosage), 4 h (time), 50 mL (volume), 200 mg/L (OXAPN concentration), and 20 °C (temperature)). According to the experiments, KNs and β-CD/KNs had significantly higher OXAPN-loading characteristics as the pH increased from pH 2 (6.7 mg/g for KNs and 16.3 mg/g for β-CD/KNs) to pH 8 (92.7 mg/g for KNs and 129.6 mg/g for β-CD/KNs) (Figure 5A). This was attributed to the enhancement in OXAPN’s solubility and mobility in acidic conditions [4,15]. Furthermore, [Pt(dach)(H_2_O)Cl]^+^ and [Pt(dach)(H_2_O)_2_]^2+^ are the most prevalent and persistent ionized forms developed whenever the OXAPN medication is dissolved at these acidic pH values [16]. Therefore, the positively charged OXAPN ions have strong electrostatic repulsion behaviors with the hydrogenated chemical structures of KNs and β-CD/KNs that are enriched by many positively charged hydronium ions [41]. Therefore, during the loading of OXAPN into KNs and β-CD/KNs, the basic condition is preferred, which is in accordance with the detected pH_(PZC)_ values of KNs (pH = 6.8) and β-CD/KNs (pH = 5.7).

##### Encapsulation Interval

The loading characteristics of both KNs and β-CD/KNs in terms of duration are vital factors in establishing the equilibrium state of the OXAPN-loading reactions as well as regulating the loaded amount according to the stated dosage. Experiments were conducted through a range of loading intervals from 1 h to 24 h, while other factors (carrier dosage, pH, volume, temperature, and OXAPN concentration) were held constant at 25 mg, 50 mL, 20 °C, and 200 mg/L, respectively. The OXAPN-encapsulating qualities of KNs and β-CD/KNs show a sufficient increasing impact for the increase in the test period of up to 18 h for KNs and 14 h for β-CD/KNs (Figure 5B). After that, the experimentally determined loading rate remains steady or hardly varies, and the amount of OXAPN that is loaded does not significantly increase (Figure 5B). This demonstrates that the carriers are in their equilibrium states with loading capacities of 148.5 mg/g (KNs) and 159.3 mg/g β-CD/KNs). Since there were numerous active sites on the outer surfaces of the KNs and β-CD/KNs at the start of the tests, OXAPN was actually encapsulated at a considerably faster rate [42]. As the loading time was prolonged, more sites were filled by OXAPN, which caused the total number of available sites to progressively decline and the loading rate to slow down. The complete occupancy of such active sites resulted in an equilibrium state in which no further OXAPN molecules were able to be loaded [43].

##### OXAPN Concentration

The OXAPN-loading properties of KNs and β-CD/KNs with regard to the investigated OXAPN concentration significantly establish their maximum capacities, their equilibrium properties, and the control of the entrapped OXAPN dosage. The effect of OXAPN concentrations varied from 100 to 800 mg/L after specific adjustments of the influencing factors (25 mg (carrier dosage), pH 8, 20 °C (temperature), 50 mL (solution volume), and 24 h (duration)). The detectable encapsulation properties of KNs and β-CD/KNs are improved by high OXAPN concentrations (Figure 5C). High OXAPN concentrations increase the driving forces as well as diffusion characteristics of dissolved OXAPN ions, which increases the probability of their interaction with the functional loading sites and, as a result, the OXAPN-loading capacities of KNs and β-CD/KNs [36,44]. This improvement effect could be detected up to an OXAPN concentration of 500 mg/L for KNs and 600 mg/L for β-CD/KNs. After that, an increase in OXAPN concentration resulted in a negative influence, indicating an equilibrium condition (Figure 5C). As a consequence, these concentrations (500 mg/L for KNs and 600 mg/L for -CD/KNs) represent the saturation level of KNs and β-CD/KNs at which they accomplish their actual greatest loading capacities (302 mg/g for KNs and 348 mg/g for β-CD/KNs) (Figure 5C). The mentioned increase in the surface area after the β-CD functionalization process, as well as the organophilic characteristics of the β-CD/KNs in contrast to hydrophilic KNs that promote their affinity to OXAPN, can explain the detected higher OXAPN capacity of β-CD/KNs than KNs. Additionally, there was a notable increase in the number of existing encapsulation sites after the β-CD hybridization processes.

##### Effect of Temperature

The drug-loading experiments were performed with a steady increase in temperature between 20 and 60 °C to assess whether the temperature impacted the OXAPN-loading capacities of KNs and β-CD/KNs either positively or negatively (Figure 4D). The loading duration was set at 24 h, the OXAPN concentration was 800 mg/L, the volume was 50 mL, the carrier dose was 20 mg, and the temperature was 20 °C. The reduction in OXAPN-loaded amounts with increasing temperature (Figure 5D) demonstrates the exothermic behavior of KNs and β-CD/KNs’ loading processes. The loading capacities of KNs and β-CD/KNs at 60 °C are 257.4 mg/g and 322.4 mg/g, respectively (Figure 5D). Based on the results of the loading tests, both KNs and β-CD/KNs exhibit desirable characteristics as OXAPN carriers because of their significant loading qualities. Also, the quantities of the entrapped medication can potentially be controlled on both KNs and KNTs by adjusting the loading variables, such as pH, loading time, OXAPN concentration, and temperature.

#### 3.2.2. Encapsulation Mechanism

##### Kinetic Properties

Intra-Particle Diffusion Behavior

**Figure 6:**
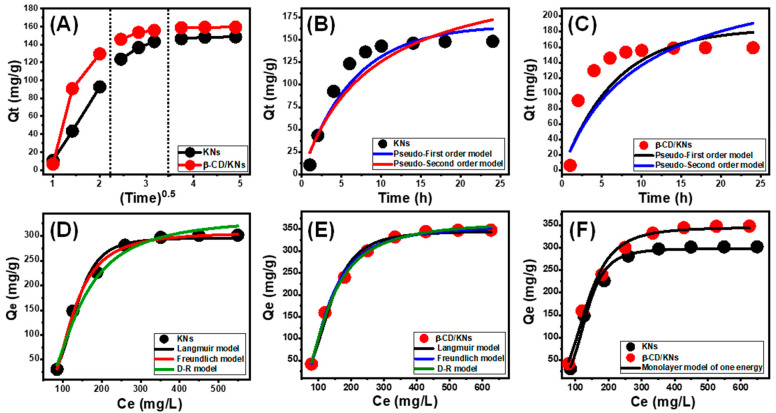
The intra-particle diffusion curves of OXAPN–loading processes by KNs and β-CD/KNs (**A**), the fitting of the OXAPN–loading reactions by KNs with kinetic models (**B**), the fitting of the OXAPN-loading reactions by β-CD/KNs with kinetic models (**C**), the fitting of the OXAPN-loading reactions by KNs with classic isotherm models (**D**), the fitting of the OXAPN-loading reactions by β-CD/KNs with classic isotherm models (**E**), and the fitting of the OXAPN-loading reactions by KNs and β-CD/KNs with advanced isotherm model (**F**).

For OXAPN-loading processes into both KNs and β-CD/KNs (Figure 5A), three-stage intra-particle diffusion curves were observed, with no crossovers between the curves’ initiation points. This demonstrates the OXAPN’s encapsulation via cooperative processes, together with the significant effect of ion diffusion in the direction of the KNs and β-CD/KNs’ active receptors [44,45]. This might include (A) loading by the dispersed active sites across the external surface (border), (B) intra-particle diffusion, and (C) the mechanistic influence of the equilibrium states [46]. In the beginning stages of the tests, the existence of the first stage indicates the activation of the exterior loading mechanisms, and the number of surface-active sites regulates the effectiveness of the encapsulation processes (Figure 6A) [47]. A new stage was noticed by increasing the encapsulation duration (Figure 6A), which indicates the presence of different mechanisms, such as the influence of the OXAPN diffusion reactions and the layered encapsulating activities. The third stage was identified as the most predominant stage by attending to the equilibrium states of the KNs and β-CD/KNs during the OXAPN-loading processes. This suggests that all of the effective binding sites were filled up or devoured by the OXAPN ions (Figure 6A) [5,44]. The encapsulation activities at this stage are influenced by different mechanisms, including interionic attraction and molecular interaction [36].

Kinetic Modeling

The kinetic characteristics of the OXAPN-encapsulating activities accomplished by the KNs and β-CD/KNs were assessed based on the pseudo-first-order (PFO) (Equation (3)) and pseudo-second-order (PSO) (Equation (4)) models. This was accomplished by non-linearly fitting the obtained results to the descriptive equations of these two models, based on the coefficient of correlation (R^2^) and chi-squared (χ^2^) values as indications of the fitting degree (Table 1; Figure 6B,C).

(4) $$Q_{t\;}=Q_{e}\;\left(1-e^{{-k}_{1}.t}\right)$$

(5) $$Q_{t}=\frac{Q_{e\;}^{2}k_{2}t}{1+Q_{e}k_{2}t}$$

Based on the determined values of R^2^ and χ^2^, OXAPN was loaded into KNs and β-CD/KNs in accordance with the kinetic criteria of the PFO model in comparison to the kinetic properties of the PSO model (Table 1). This was supported by the significant matching between the experimentally detected loading capacity values (148.5 mg/g (KNs) and 159.3 mg/g (β-CD/KNs)) and the theoretically computed values as parameters of the representative PFO model (166.6 mg/g (KNs) and 184.3 mg/g (β-CD/KNs)). These kinetic specifications indicate the dominant effect of the present physical OXAPN-loading mechanisms, which may involve electrostatic attractions [48,49]. However, the observed match of the OXAPN-loading experiments by KNs and β-CD/KNs with PSO kinetics at reasonable degrees displays the important role of some faint chemical processes, either essential or as support mechanisms. This could include the formation of a chemical complex in addition to electron exchanges, hydrogen bonds, and electron-sharing processes [36,48]. A combination of both physical and chemical mechanisms may result from the physical encapsulation of OXAPN molecules above an outermost layer of chemically loaded drug molecules [50].

##### Isotherm Properties

Classic Isotherm Models

The equilibrium characteristics of the OXAPN-loading reactions into KNs and β-CD/KNs as potential carriers were illustrated using the conventional assumptions of Langmuir (Equation (5)), Freundlich (Equation (6)), and Dubinin–Radushkevich (D-R) (Equation (7)) by non-linearly fitting the results with the models’ illustrative equations while considering the correlation coefficient (R^2^) and chi-squared (χ^2^) values as indicators of the fitting degree (Table 1; Figure 6D,E).

(6) $$Q_{e}=\frac{Q_{max}\;bC_{e}}{\left(1+bC_{e}\right)}$$

(7) $$Q_{e}=K_{f}C_{e}^{1/n}$$

(8) $$Q_{e}=Q_{m}e^{-\beta \varepsilon^{2}}$$

The encapsulation of OXAPN into KNs and β-CD/KNs displays the equilibrium behaviors of the Langmuir isotherm instead of the Freundlich hypothesis in accordance with the established values of the model-fitting parameters. As a result, the OXAPN molecules were uniformly encapsulated on the exterior surfaces of the KNs and β-CD/KNs in monolayer forms by numerous homogenously dispersed active receptors [5,47]. Additionally, the values of the RL parameter being less than one reveal the favorable encapsulation of OXAPN ions into the KN and β-CD/KNs carriers. Additionally, the theoretical maximal OXAPN encapsulation capacities of KNs and β-CD/KNs were estimated as mathematical parameters of the Langmuir isotherm to be 309.3 mg/g and 362.4 mg/g using the Langmuir fitting parameters (Table 1).

Regarding the studied D-R model, its isotherm characteristics might significantly reveal the energetic heterogeneity of KNs and β-CD/KNs as carriers of OXAPN, whether they have homogeneous or heterogeneous surfaces [51]. Determining the Gaussian energy (E) as an attained theoretical parameter of the D-R model considerably emphasizes the nature of the predominant loading mechanisms, whether they have chemical or physical characteristics. While the chemical loading system displays values >16 KJ/mol, the physical loading reaction shows a Gaussian energy of less than 8 KJ/mol. Gaussian energy levels between 8 and 16 KJ/mol are indicative of complicated systems or weak chemical loading processes [5,51]. The OXAPN encapsulation processes by KNs and β-CD/KNs have corresponding Gaussian energies of 4.65 kJ/mol and 4.38 kJ/mol, respectively (Table 1). These values reflect the encapsulation of OXP by physical mechanisms, and the lower E value of β-CD/KNs than KNs validates the effect of the β-CD in reducing the formation of chemical complexes or hydrogen bonding with the structure of kaolinite sheets and inducing electrostatic attraction processes.

Advanced Isotherm Models

The advanced isotherm models selected, which depend on the equilibrium basics of statistical physics theory, provide more insight into KNs and β-CD/KNs as OXAPN carriers in terms of the interface between the medication in solution and the carrier surfaces. The loading characteristics and their controlled mechanistic activities were investigated using an advanced monolayer model with one energy site (Equation (9)) and associated mathematical variables, either steric or energetic (Figure 6F; Table 1). The root mean square error (RMSE) and the determination coefficient (R^2^) were considered to be the main factors determining the fitting degrees.

(9) $$Q=nN_{o}=\frac{nN_{M}}{1+{\left(\frac{C1/2}{Ce}\right)}^{n}}=\frac{Q_{o}}{1+{\left(\frac{C1/2}{Ce}\right)}^{n}}$$

The model’s derived steric mathematical characteristics included the occupied active receptor density (Nm _(OXAPN)_) of the KNs and β-CD/KNs, the number of OXAPN ions that were loaded per each active site (n _(OXAPN)_), and the OXAPN encapsulation capacity of the KNs and β-CD/KNs at their saturation state (Qsat _(OXAPN)_). The determined encapsulating energy (E) was determined as the energetic parameter. The chemical modification of the separated kaolinite nanosheets (KNs) into β-CD/KNs led to an increase in the density of the effective encapsulation site from 66.3 mg/g (KNs) to 98.7 mg/g for β-CD/KNs. This might be due to the integration of further active functional groups related to β-CD or the increase in the interaction interface with a noticeable increase in surface area. This was reflected in the estimated OXAPN-loading capacities of the KNs and β-CD/KNs in their saturation states, which were considerably increased after the modification procedures, from 304.9 mg/g for KNs to 355.3 mg/g for β-CD/KNs. Furthermore, the identified numbers of the predicted loaded OXAPN ions per each active site of the KNs and β-CD/KNs (n _(OXAPN)_) reveal an essential effect of the modification process on their surface characteristics as carriers. In the course of loading OXAPN into KNs and β-CD/KNs, the theoretical values of n _(OXAPN)_ are 4.7 and 5.85, respectively. These numbers are greater than 1, which promotes the vertical loading of these ions on the exteriors of KNs and β-CD/KNs as well as the subsequent absorption of them by means of multi-molecular processes [52,53]. However, each active site of a KN can hold up to six molecules of OXAPN, whereas β-CD/KNs can hold up to five molecules of OXAPN at each active site. Based on the theoretically determined residual OXAPN concentrations at the half saturation states (C1/2) and its solubility in water, the loading energies (E) were estimated using Equation (10) (Table 1).

(10) $$∆E=-RT\;ln\left(\frac{S}{C_{1/2}}\right)$$

The determined loading energies of OXAPN into KNs and β-CD/KNs are −7.5 KJ/mol and −6.4 KJ/mol, respectively. These values support the previous findings about the physical encapsulation mechanisms (Δ*E* ≤ 40 kJ/mol) of OXAPN into KNs and β-CD/KNs [52]. These processes might involve van der Waals forces (Δ*E* = 4 to 10 kJ/mol), dipole forces (Δ*E* = 2 to 29 kJ/mol), and hydrogen bonding (Δ*E* < 30 kJ/mol) [28,54].

##### Thermodynamic Properties

**Figure 7:**
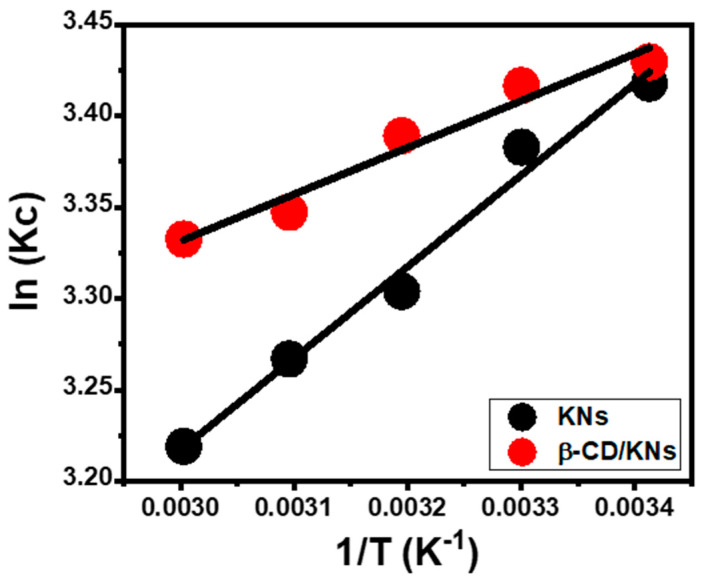
The fitting of the OXAPN-loading results into KNs and β-CD/KNs with the Van’t Hof thermodynamic equation.

The thermodynamic properties of the OXAPN-encapsulating mechanisms by KNs and β-CD/KNs were investigated in an ambient temperature range of 20 °C to 60 °C. This was carried out considering the other experimental variables at precise values (25 mg (dosage), 24 h (time), 50 mL (volume), 800 mg/L (OXAPN concentration), and pH 8). This includes fundamental thermodynamic functions including Gibbs free energy (Δ*G*°) (Equation (12)) in addition to entropy (Δ*S*°) and enthalpy (Δ*H*°) that were obtained by fitting the data to the Van^,^t Hof equation (Equation (12)) (Figure 7) [27].

(11) $$In\left(K_{c}\right)=\frac{{∆S}^{o}}{R}-\frac{{∆H}^{o}}{RT}$$

(12) $${∆G}^{0}=-RT\;In\;K_{c}$$

Recognizing the values of Δ*S*° and Δ*H*° with negative signs demonstrates the exothermic, spontaneous, and favorable characteristics of the OXAPN encapsulation mechanisms using KNs and β-CD/KNs as potential carriers (Table 1). Additionally, the positively signed Δ*S*° values of the KNs and β-CD/KNs loading systems for OXAPN indicated an increase in the randomness of the reactions that occurred with regard to the temperature that was being tested (Table 1).

### 3.3. In Vitro Release Profiles

**Figure 8:**
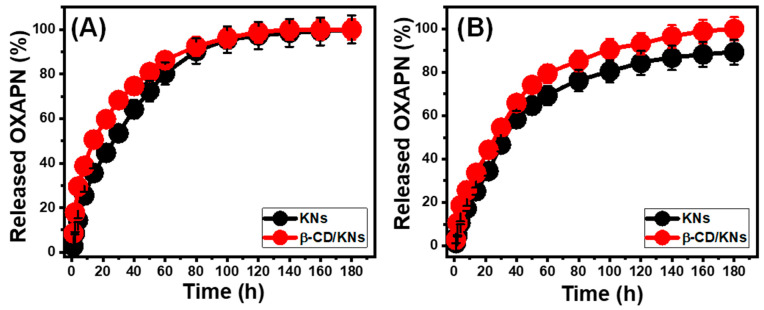
The OXAPN release profiles of KNs and β-CD/KNs either in acetate buffer (**A**) or phosphate buffer (**B**).

For monitoring the release patterns of KNs and β-CD/KNs, the percentages of the diffused OXAPN into the two assessed buffer solutions (phosphate (pH 7.4) and acetate (pH 5.5)), which acted as media to imitate the examined tumor cells, were determined (Figure 8). The observed OXAPN release patterns of KNs and β-CD/KNs into the evaluated buffers indicate considerable differences in diffusion rates when the release duration is extended. In the early diffusion phases of the experiments, the observed release parentages supported rapid diffusion rates. These rates gradually decline until an equilibrium duration with a stable rate of diffusion or the complete release of the encapsulated OXAPN dose occurs (Figure 8). The notable rapid diffusion of OXAPN from KN and β-CD/KNs structures throughout the early phases of the experiments was mainly explained by the desorption processes of the physically adsorbed OXAPN ions or the poorly bonded ions to the surface-active groups of the KNs and β-CD/KNs [19,55]. The release mechanisms were then restrained mostly to the chemically bonded drug ions with the active groups of the KNs and β-CD/KNs, leading to a drop in release rates [12,27]. At this point, all of the faintly charged OXAPN ions had completely diffused. The OXAPN release percentage in the investigated acetate buffering solution (pH 5.5) is greater than the displayed release percentage in the phosphate-buffered solution (pH 7.4) (Figure 8). Such release properties were attributed to OXAPN’s superior mobility and solubility in low-pH fluids [15].

In phosphate and acetate buffers, OXAPN is generally released from KNs over a period of 180 h without the complete diffusion state being observed. Within 30 h (pH 5.5) and 40 h (pH 7.4), over 50% of the loaded dose of OXAPN was released. The greatest diffusing percentages that were actually achieved were 100% (180 h; pH 5.5) and 89.3% (180 h; pH 7.4) (Figure 8A). Loaded OXAPN doses within the β-CD/KNs framework exhibit better diffusion characteristics as compared to KNs. About half of the loaded dose of OXAPN diffused after 14 h (pH 5.5) and 30 h (pH 7.4). The full diffusion state was attained after 140 h (pH 5.5) and 180 h (pH 7.4) (Figure 8B). The effect of the β-CD functionalization technique on the release characteristics of the final composite (β-CD/KNs) is reflected in the elevated OXAPN diffusion features. The integrated β-CD as a modified surface considerably limits the available opportunities for forming hydrogen bonds or chemical complexes between the OXAPN structure and the siloxane groups of the KNs. Additionally, the structure acquires more free active sites from the β-CD-modified surface, which increases the release rates and efficiency of their physically bonded OXAPN ions [13,55,56].

When treating tumor cells, it is recommended to continuously and slowly administer OXAPN molecules as anticancer chemotherapy because this method allows for prolonged contact and substantive interaction between the malignant cells and the administered therapy [7,12]. In certain situations, it is advisable to provide the necessary therapeutic dosage of the drug at certain times by delivering it quickly, abruptly, and over a short period of time. Therefore, the well-established delivery systems of KNs and β-CD/KNs are particularly effective for loading and releasing OXAPN in controlled quantities.

### 3.4. Release Kinetic Studies

The kinetic features of the OXAPN-releasing reactions from KNs and β-CD/KNs can potentially be used as evidence of the mechanism that operates during the diffusion reactions. The explored kinetic releasing models include the zero-order (Z-O) (Equation (13)), first-order (F-O) (Equation (14)), Higuchi (H-G) (Equation (15)), Hixson-Crowell (H-C), and Korsmeyer–Peppas (K-P) models [12]. These models were evaluated based on the outcome of the linear regression fitting methods of the release data and their mathematical equations within the two buffers under consideration.

(13) $$W_{t}-W_{0}=K_{0}.t\;$$

(14) $$\ln\left(\;W_{\infty }/W_{t}\right)=K_{1}.t\;$$

(15) $$W_{t}=K_{h}t^{1/2}$$

(16) $$W_{o}^{1/3}-W_{t}^{1/3}=K_{HC}t$$

(17) $$W_{t}/\;W_{\infty \;}=K_{p\;}t^{n}$$

According to the Z-O model’s assumption, OXAPN release might occur without any particular influence of the loaded dosage on the release profiles, and the system implies a constant diffusion rate [13]. According to the F-O hypothesis, the quantity of OXAPN molecules loaded has a significant impact on the release properties [1]. For the kinetic features that fit the Higuchi kinetics principle, OXAPN is released through diffusion processes that are also affected by certain factors [1,57]. They include the following: (1) loaded OXAPN releases at constant rates but only in one direction; (2) the loaded dose is greater than the diffused rate; (3) the solubility and swelling properties of the used carrier have no effect on the effectiveness of the release; and (4) the carrier has a clear sink nature [13]. Hixson-Crowell kinetics are used to illustrate release systems comprising the operation of erosion mechanisms and exhibiting a controlling impact of the surface area as well as the particle diameter of the carriers on the efficiency of the reactions [13,58]. The Korsmeyer–Peppas model largely illustrates release mechanisms that integrate the cooperation of diffusion and erosion processes, notably for the hybrid delivery system [1,59].

**Figure 9:**
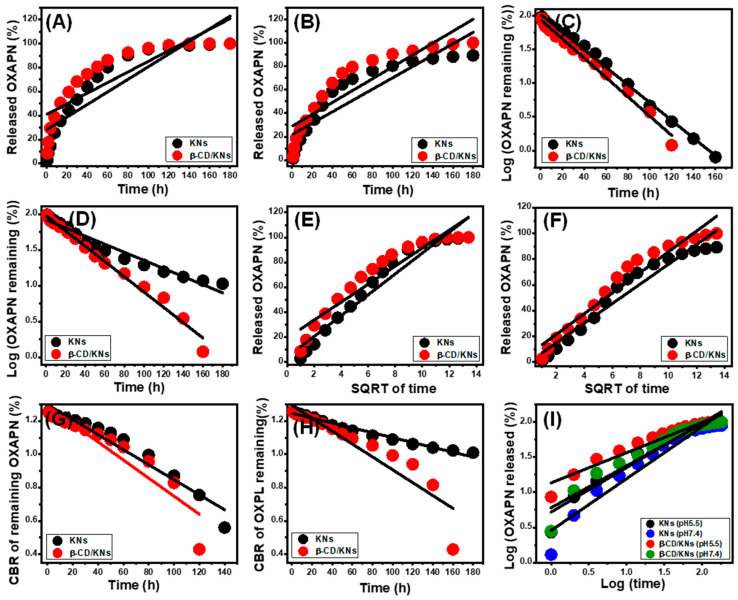
The fitting of the OXAPN release results with the zero-order model (**A**,**B**), the fitting of the OXPN release results with the first-order model (**C**,**D**), the fitting of the OXAPN release results with the Higuchi model (**E**,**F**), the fitting of the OXAPN release results with the Hixson–Crowell model (**G**,**H**), and the fitting of the OXAPN release results with the Korsmeyer–Peppas model (**I**).

The determined fitting degrees (depending upon the obtained determination coefficient) show that within both the acetate- and phosphate-buffered solutions, the OXAPN release properties of the KNs and β-CD/KNs follow the kinetic characteristics of the F-O model (Figure 9C,D; Table 1) better than the Z-O model (Figure 9A,B; Table 1). According to this kinetic behavior, both KNs and β-CD/KNs’ release properties are significantly influenced by the overall quantity of loaded OXAPN medicine. The KNs and β-CD/KNs’ release results are consistent with the Higuchi (Figure 9E,F; Table 1) and Hixson-Crowell model (Figure 9G,H; Table 1) kinetic hypotheses. Therefore, the OXAPN release characteristics of KNs and β-CD/KNs included a mix of diffusion and erosion processes. The partial disintegration of the β-CD polymeric matrix and silicate minerals at higher pH values could potentially be the cause of the erosion mechanism. The strong agreement between the OXAPN release patterns and the kinetic concepts of the Korsmeyer–Peppas model demonstrates the prevalence of diffusion processes as the main mechanism, in addition to the accelerating impact of erosion processes (Figure 9I; Table 1). The computed diffusion exponent (n) as an establishing parameter for KNs (0.62 (acetate) and 0.72 (phosphate)) and β-CD/KNs (0.49 (acetate) and 0.59 (phosphate)) confirm non-Fickian transport behavior, which is in agreement with previous kinetic findings on the collaboration of both diffusion and erosion mechanisms [42].

### 3.5. Cytotoxicity Properties

**Figure 10:**
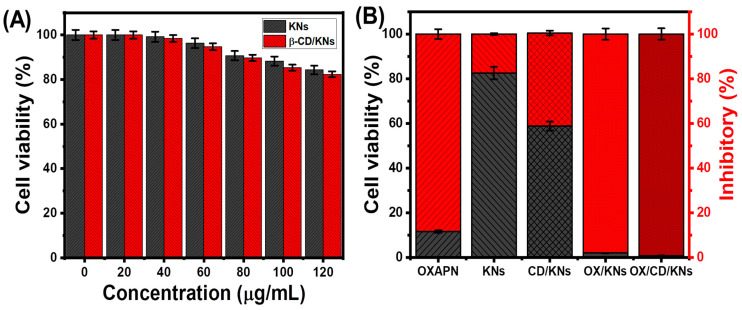
The cytotoxicity effect of free KNs and β-CD/KNs on the CCD-18Co cell lines (**A**) and the cytotoxic effect of OXAPN, free KNs, free β-CD/KNs, OXAPN-loaded KNs, and OXAPN-loaded β-CD/KNs on colorectal cancer cells (HCT-116) (**B**).

The cytotoxicity of free KNs and β-CD/KNs, as well as their OXAPN-loaded compounds, was evaluated in colorectal cancer (HCT-116) and normal colorectal fibroblast (CCD-18Co) cell lines. According to the established cytotoxicity effects on normal cells, both free KNs and β-CD/KNs demonstrate notable safe and biologically compatible properties within the examined dosage range (20 to 120 µg/mL). At 120 µg/mL dosages, the percentages of cell viability in the presence of KNs and β-CD/KNs were 84.3% and 82.3%, respectively (Figure 10A). Free KNs and β-CD/KNs show considerable cytotoxic impacts on the HCT-116 malignant cell lines, especially at high doses (>50 µg/mL). Free KNs (500 µg/mL) revealed 17.41% inhibitory impact, 143.6 µg/mL IC-50, and 82.59% cell viability (Figure 10B). For free β-CD/KNs, the values found were 97.3 µg/mL (IC-50), 41.61% (inhibitory%), and 58.83% (cell viability) (Figure 10B). Such cytotoxic properties could be attributed to the considerable surface reactivity of kaolinite-separated layers and the biological activity of CD, in combination with the verified oxidation properties of clay nanomaterials as a result of the presence of transitional metal ions as structural impurities.

Compared to OXAPN alone, OXAPN-loaded KNs and β-CD/KNs exhibit a more cytotoxic impact. The determined cell viability, inhibitory percentage, and IC-50 during the incorporation of the OXAPN drug as a free drug without carriers were 11.62%, 88.38%, and 17.85 µg/mL, respectively. The cell viability, inhibitory percentage, and IC-50 values of OXAPN-encapsulated KNs (500 µg/mL) were 2.04%, 97.96%, and 15.4 µg/mL, respectively (Figure 10B). OXAPN-loaded β-CD/KNs (500 µg/mL) had a cell viability of 0.86%, an inhibitory percentage of 99.14%, and an IC50 of 1.87 µg/mL (Figure 10B). The applications of such carriers significantly increase the interaction interface between the cancer cells and the drug molecules, preserving prolonged and continuous interaction effects.

## 4. Conclusions

Raw kaolinite mineral was effectively exfoliated into separated kaolinite nanosheets, which were successfully hybridized with β-cyclodextrin biopolymer (β-CD/KNs). KNs, as well as β-CD/KNs, were assessed as promising carriers of OXAPN therapy, achieving significant loading capacities (304.9 mg/g (KNs) and 355.3 mg/g (β-CD/KNs)). The higher loading properties of β-CD/KNs, as compared to KNs, were attributed to its organophilic properties and the enrichment of its structure with more active sites (98.7 mg/g) than KNs (66.3 mg/g). Each active site of the KNs and β-CD/KNs can be loaded with more than one OXAPN molecule in vertical form and by multi-molecular mechanisms that are dominantly of a physical nature, considering both Gaussian energy (<8 KJ/mol) and loading energy (<40 KJ/mol). The detected OXAPN release profiles of both KNs and β-CD/KNs continued slowly up to 120 h, with noticeably faster properties for β-CD/KNs. This release behavior displays non-Fickian transport with cooperative diffusion and erosion mechanisms. KNs as well as β-CD/KNs as free particles exhibit considerable cytotoxic impacts on HCT-116 cancer cells (71.4% cell viability (KNs) and 58.83% cell viability (β-CD/KNs)) and strong effects after their loading with OXAPN (2.04% cell viability (OXAPN/KNs) and 0.86% cell viability (OXAPN/β-CD/KNs).

## Figures and Tables

**Table 1:** The obtained mathematical parameters of the studied kinetic, classic isotherm, advanced isotherm, thermodynamic, and release kinetic models.

Model		Parameters		KNs	CD/KNs
**Kinetic models**					
Pseudo-first-order		K_1_ (min^−1^) Qe _(Cal)_ (mg/g) R^2^ X^2^		0.155 166.6 0.94 4.1	0.194 184.3 0.86 5.7
Pseudo-second-order		k_2_ (g mg^−1^ min^−1^)		4.9 × 10^−4^	3.89 × 10^−4^
		Qe _(Cal)_ (mg/g)		234.5	267.3
		R^2^ X^2^		0.92 5.4	0.84 7.3
**Isotherm models**					
Langmuir		Q_max (mg/g)_ b(L/mg) R^2^ X^2^ RL		309.3 0.0057 0.99 3.67 0.18–0.63	362.4 0.0015 0.99 0.84 0.45–0.86
Freundlich		1/n k_F_ (mg/g) R^2^ X^2^		0.33 6.4 0.99 4.2	0.75 3.26 0.98 1.02
D-R model		β (mol^2^/KJ^2^) Q_m_ (mg/g) R^2^ X^2^ E (KJ/mol)		0.0231 337.13 0.98 1.64 4.65	0.026 368.3 0.92 0.24 4.38
Monolayer model of one energy		n Nm (mg/g) Q_(sat)_ (mg/g) ∆E (kJ/mol)		4.7 66.3 304.9 −7.5	3.6 98.7 355.3 −6.4
**Thermodynamics**					
∆G° (kJ mol^−1^)			293.13	−8.32	−8.36
			303.13	−8.52	−8.61
			313.13	−8.60	−8.82
			323.13	−8.77	−8.99
			333.13	−8.91	−9.23
ΔH° (kJ mol^−1^)				−4.16	−2.13
ΔS° (J K^−1^ mol^−1^)				14.24	21.3
**Release kinetics**					
**Models**		**Determination coefficient**			
		**KNs**		**CD/KNs**	
	**Acetate buffer (pH 5.5)**		**Phosphate buffer (pH 7.4)**	**Acetate buffer (pH 5.5)**	**Phosphate buffer (pH 7.4)**
Zero-order	0.75		0.79	0.72	0.78
First-order	0.99		0.94	0.98	0.98
Higuchi	0.92		0.93	0.90	0.94
Hixson-Crowell	0.97		0.89	0.94	0.85
Korsmeyer-Peppas	0.95		0.93	0.98	0.96
n	0.62		0.72	0.49	0.59

## Data Availability

Data are available upon reasonable, by the Corresponding Authors.
